# Sortation Control Using Multi-Agent Deep Reinforcement Learning in *N*-Grid Sortation System

**DOI:** 10.3390/s20123401

**Published:** 2020-06-16

**Authors:** Ju-Bong Kim, Ho-Bin Choi, Gyu-Young Hwang, Kwihoon Kim, Yong-Geun Hong, Youn-Hee Han

**Affiliations:** 1 Department of Computer Science and Engineering, Korea University of Technology and Education, Cheonan 31253, Korea; jubong1992@gmail.com (J.-B.K.); chb3350@koreatech.ac.kr (H.-B.C.); to6289@koreatech.ac.kr (G.-Y.H.); 2 Department of Knowledge-Converged Super Brain Convergence Research, Electronics and Telecommunications Research Institute, Daejeon 34129, Korea; kwihooi@etri.re.kr (K.K.); yghong@etri.re.kr (Y.-G.H.)

**Keywords:** sortation system, n-grid sortation system, reinforcement learning, multi-agent reinforcement learning

## Abstract

Intralogistics is a technology that optimizes, integrates, automates, and manages the logistics flow of goods within a logistics transportation and sortation center. As the demand for parcel transportation increases, many sortation systems have been developed. In general, the goal of sortation systems is to route (or sort) parcels correctly and quickly. We design an *n*-grid sortation system that can be flexibly deployed and used at intralogistics warehouse and develop a collaborative multi-agent reinforcement learning (RL) algorithm to control the behavior of emitters or sorters in the system. We present two types of RL agents, emission agents and routing agents, and they are trained to achieve the given sortation goals together. For the verification of the proposed system and algorithm, we implement them in a full-fledged cyber-physical system simulator and describe the RL agents’ learning performance. From the learning results, we present that the well-trained collaborative RL agents can optimize their performance effectively. In particular, the routing agents finally learn to route the parcels through their optimal paths, while the emission agents finally learn to balance the inflow and outflow of parcels.

## 1. Introduction

The intralogistics industry is one of the fastest growing industries driven by the automation of operations and the digitization of overall process procedures. Logistics is the process of planning and organizing to make sure that resources are in the places where they are needed, while intralogistics is the management and optimization of production and distribution processes in an internal place such as a warehouse. Logistics is how to move products from one point to another point, and intralogistics is the same concept, but more related to how to get products most efficiently from the receiving place to the shipping place in a warehouse, a factory, or a plant [1,2,3]. The globalization and high demand of electronic commerce cause increasingly complex material handling systems, while market predictions are less reliable [4,5]. Therefore, a material handling system must be able to categorize and transport products of various types and sizes and be able to adapt to changing needs [6].

The most basic material handling system in intralogistics is a sortation system, which sorts, routes, consolidates, and converts a wide range of parcel types to specific destinations [5]. The indicators of the performance of the sortation system are (1) accuracy (i.e., how correctly the parcels are classified according to their destination) and (2) throughput (i.e., how fast the parcels are classified).

Recently, information and communication technology (ICT) has been further developed based on deep learning, and reinforcement learning (RL) optimizes the control behavior of the target system of interest by developing the intelligence of the agent [7,8]. It is based on the reward information from the behavioral perspective of the target system. Reinforcement learning has been widely used beyond games, go and chess, and is now widely used for optimal control in drones and autonomous cars [9,10]. In particular, it has been also used for optimal control of IoT devices, machines, and robots in smart factories [11]. On the other hand, research using RL in multi-agent systems has been done, and several studies have proven that the system outperforms the artificial control formula made by humans [12,13]. In a multi-agent system, agents interact with and adapt to the given complex and dynamic system. In situations where agents can cooperate with other agents, it has been reported that they can adapt and learn more quickly [14,15,16,17]. In particular, the work in [18,19] presented smart factory frameworks controlled by multiple agents assisted with big data based feedback and service-oriented communication.

In a smart factory environment, intralogistics companies have tried to improve sortation task efficiency and reduce human costs through the automation of sortation systems. However, this automation system still requires the design of conveyor layouts, and any coding (or modification) of automation programs is inevitable [5,20,21]. In fact, a sortation system has multiple objectives: (1) parcels should be correctly classified to the specific destinations; (2) congestion should not occur within the sortation system in order to sort the parcels in a short time; and (3) a kind of deadlock or collision between routing (or sorting) modules should be avoid. In response, GEBHARDT Intralogistics Group and Karlsruhe Institute of Technology (KIT) developed GridSorter [22], which provides a planning optimization to the sortation functions of the conveying components in a rectangular grid plane (e.g., a kind of chessboard made of conveyor modules).

In planning, however, it is humans that invent the strategies (e.g., Dijkstra’s algorithm) to optimize the performance of a sortation system, based on a model of the given sortation system. A model of a sortation system is too complex for humans to design the planning strategies completely, so that it leads to the model being simple and avoiding complex tasks. Planning may not be possible to make strategies to cope with unexpected situations in a sortation system.

In this paper, we propose a new flexible sortation system, called the *n*-grid sortation system. Its design concept is similar to GridSorter, but in the *n*-grid sortation system, autonomous agents control each module by themselves. We also propose a multi-agent deep RL algorithm to optimize the multi-objective control of the sortation tasks. The objective of an RL algorithm is to maximize the expected cumulative rewards through interaction with the target system. However, interaction with real target systems is time-consuming and vulnerable to errors and malfunctions. Therefore, we implement a virtual version of the proposed *n*-grid sortation system using the 3D factory simulator called FACTORY I/O 2.0 [23] and verify the proposed multi-agent RL algorithm through real-time interaction with the virtual version. The most prominent feature of this simulator is that it can be used with several types of real PLCsor microcontrollers. In addition, it provides a full-fledged I/O driver for interacting with an external controller, so that a deep learning program in a PC can receive any sensor information from parts of the simulator and control them directly. In this paper, that is, we intend to train the RL agents within the virtual system, transfer them into a real system, and then, use them without modification or with a little extra training. We think that the intention of this strategy is similar to that of trying to build a cyber-physical system (CPS).

The contributions of this paper can be summarized as follows:

1. We propose the design of a compact and efficient sortation system called the *n*-grid sortation system.
2. We present a cooperative multi-agent RL algorithm to control the behavior of each module in the system.
3. We describe how the RL agents learn together to optimize the performance of the *n*-grid sortation system.

The remainder of this paper is organized as follows. In Section 2, we review the sortation system and deep RL technology that motivate our research work. In Section 3, we describe the overall system architecture and procedure of the proposed cooperative multi-agent *n*-grid sortation system. In Section 4, we present our cooperative multi-agent RL algorithm to control each module in the proposed system. In Section 5, we evaluate the performance of the proposed algorithm on the system and present that the well-trained collaborative RL agents can optimize their performance effectively. Finally, we provide concluding remarks and future work in Section 6.

## 2. Related Work

In this section, we introduce the sortation system and its design goals and explain the contribution and the limit of the related work. We also explain the deep RL technology to solve the problem presented in this work.

### 2.1. Sortation Systems

Sortation is defined as the process of identifying items in a (conveyor) system and diverting them to specific destinations. The sortation system (or sorter) is a system of sorting, routing, consolidating, and diverting a wide range of types of parcels to specific destinations [24].

The conventional sortation systems have been designed with conveyor based ring or line layouts. Since the layout design of the sortation system is directly related to throughput, the study of it has been done for decades [25,26,27]. However, the design of the sortation system of the previous studies could not be modularized and decentralized, and this makes it difficult to adapt to small changes with diverse requirements.

GridSorter [22] consists of a connected area of 90 degree converters that can change the parcel’s conveying direction into the desired direction to east, west, south, and north. (How GridSorter works can be seen in the YouTube video https://www.youtube.com/watch?v=Ayt2QMOfW0M). GridSorter lays out the sorting components in a rectangular grid, and its compact layout like a chessboard leads to higher throughput than conventional sortation systems and also requires less area. In addition, the converters connected in the form of a square grid are modular, so they can have different layouts depending on the requirements. It also provides the following advantages: (1) optimization of the planning of sortation functions and (2) easy adjustment of the layout according to new requirements. A plug-and-play concept is also presented to allow quick replacement of faulty modules.

### 2.2. Existing Optimization Work for the Sortation Task

There have been two approaches to optimize the sortation task in sortation systems: (1) optimal layout design and (2) optimal parcel routing scheme. Chen et al. [27] also focused on enhancing the efficiency of parcel sortation operations through optimal layout design. They proposed a simulation optimization approach to solve the shortcut design of the sortation system that could increase the processing capacity. Seibold et al. [4] provided the layout analysis prior to simulation experiments to give insights about the sorting performance of a specific layout and also presented a new decentralized routing control algorithm to reduce the average path length of parcels. However, their routing rules depended largely on the deployed system layout and the parameters of the algorithm. This means that minor changes to the layout or the parameters would have a significant impact on routing rules and performance. Westbrink et al. [28] presented the mechatronic design, simulation, and control of peristaltic movement machines for transport and sortation of parcels. By using the proposed simulation system of peristaltic movement machines, they proposed a new RL algorithm while defining the state and action sets and the rewards obtained during action execution, so that the proposed agent learned the fastest route to the parcel. Although the inherent flexibility of the RL algorithm was high, the proposed reward setting and algorithm were very simple, so the applicability of the proposed RL algorithm was rather low. In this paper, we propose a new cooperative multi-agent RL algorithm for the optimal parcel routing on the flexible grid layout of the sortation system.

### 2.3. Deep Reinforcement Learning

The optimal policy should be inferred by the trial-and-error of agents interacting with the target system. The common model of RL is the Markov decision process (MDP) [29]. In MDP, the set of the target system’s states is *S*, and state *s* at time step *t* is expressed $s_{t}\in S$. The set of agents’ actions is *A*, and the agent action *a* at time step *t* is expressed as $a_{t}\in A$. When an agent selects and performs an action $a_{t}$ at the state $s_{t}$, the target system’s state is changed to $s_{t+1}$, and the agent receives an immediate reward $r_{t+1}$. Finally, the policy π is a mapping from states to probabilities of selecting each possible action. If the agent is following a policy π at time step *t*, then π(a|s) is the probability that $a_{t}=a$ if $s_{t}=s$. The most important information for an agent to update its policy is the reward given by the target system. The goal of RL is to find an optimal policy to maximize the expected cumulative reward, incurred from time step *t*, defined by $E\left[G_{t}\right]=E\left[\sum_{k=0}^{\infty }\gamma^{k}R_{t+k+1}\right]$, where γ∈(0,1] is the discount factor.

There are two main approaches to achieve the goal of RL: (1) value based methods and (2) policy based methods. Q-learning [30] is a traditional approach to learning an optimal policy by the value based method. For a given policy π, the state-action value function is defined as the expected return conditioned on a state and action $q_{\pi }\left(s,a\right)=E_{\pi }\left[G_{t}|S=s,A=a\right]$. The agent iteratively updates the state-action value function based on samples from the target system using the following update rule:

(1) $$Q\left(S_{t},A_{t}\right)\leftarrow Q\left(S_{t},A_{t}\right)+\alpha \left[R_{t+1}+\gamma \max_{a^{\prime }\in A}Q\left(S_{t+1},a^{\prime }\right)-Q\left(S_{t},A_{t}\right)\right]$$

where α denotes the step size (or learning rate) of the update. The optimal policy is greedily determined through $arg\max_{a}Q\left(s,a\right)$ for each state *s* after sufficient learning with enough samples.

Due to the numerous state spaces in many real-world target systems, it is intractable to learn a state-action pairing for the entire MDP. Instead, we learn an approximation to the true function $q_{\pi }$. The deep Q-network (DQN) [31], one of the most well-known value based methods, approximates the state-action value function such that $Q\left(s,a;\theta \right)∼q_{\pi }\left(s,a\right)$, where θ denotes the weights of a deep learning network model.

In DQN, the experience replay memory is set up and used to store the transitions $\left(S_{t},A_{t},R_{t+1},S_{t+1}\right)$ that the agent experiences, allowing the agent to reuse these data later for optimizing the state-action value function. By sampling from it randomly at the optimization task, the agent can get a batch where transitions are decorrelated. It has been shown that this random batch greatly stabilizes and improves the DQN training performance. In addition to that, the agent also keeps a separate target state-action value function $\hat{Q}\left(s,a;\hat{\theta }\right)$ for added stability. This target function is used to generate the target *Q* values that will be used to compute the loss for every action during optimization. The issue is that at every training, the *Q* network’s weights shift, and if an agent uses a constantly shifting weights to adjust its network weights, then the weight estimations can easily spiral out of control. In order to mitigate that risk, the target function’s weights are kept frozen for most of the optimization time and only periodically or slowly updated to the primary state-action value function’s weights. In this way, the DQN training can proceed in a more stable manner.

In this paper, we set up the DQN model as (1) each routing agent working on its independent sorter and (2) each emitter agent working on its independent emitter and propose a new multi-agent RL algorithm to optimize the behavior of the sorters and emitters.

## 3. Design of the N-Grid Sortation System

This section describes the design of the *n*-grid sortation system. The system dynamics is represented by the deployed actuators and sensors. t>0 is the sampling period, in units of time. The sampling period also corresponds to the time step at which the system is running in a discrete-time manner. That is, the system receives inputs, generates output, and updates its state at sampling instants (i.e., at every time step).

The proposed *n*-grid sortation system is a decentralized and modular version of the existing sortation systems. The two performance measures of the sortation system are (1) accuracy and (2) throughput. That is, the ultimate goal of the system is to classify the parcels as accurately and quickly as possible according to their specific destinations.

**Figure 1:**
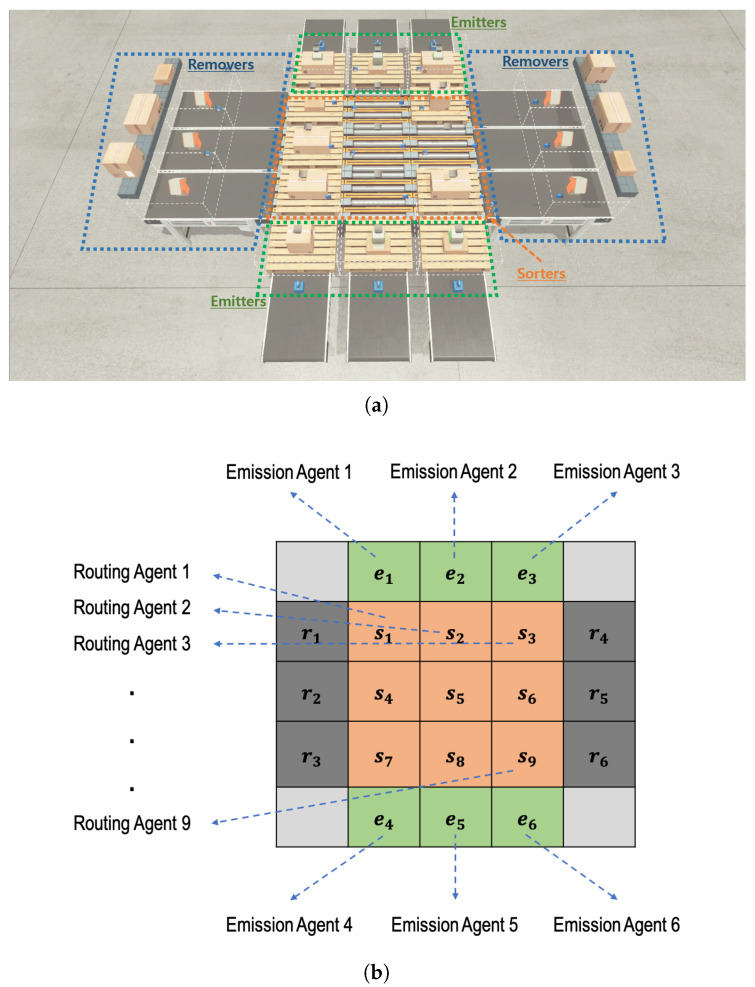
A three-gird instance of the proposed *n*-grid sortation system. (**a**) Main components of the three-grid sortation system; (**b**) Raw state representation of the three-grid sortation system.

There can be many types of parcels with different destinations in the *n*-grid sortation system. In this study, we assume that three types (e.g., (1) small:1, (2) medium:2, and (3) large:3) of parcels come into the system. As shown in Figure 1, the system consists of three main components:

- 1∼4n emitters through which parcels are fed into the sortation system,
- n×n sorters by which the incoming parcels are routed (or diverted) to their specific destination, and
- 1∼4n removers through which parcels are unloaded from the sortation system.

The sum of the numbers of emitters and removers cannot exceed 4n. One of the emitters is a starting point of a parcel, while one of the removers is a specific destination of a parcel. Each remover knows what type of parcels it should accept, and it determines whether the parcel has been correctly classified whenever it receives a parcel.

The sorters are deployed in the form of an n×n grid, and each of them is controlled by an independent routing RL agent, allowing the system to have a flexible structure. On the other hand, emitters are deployed on the side of the n×n grid, and each of them is controlled by an independent emission RL agent. The number and location of emitters are determined by the system requirements. Similarly, removers are deployed on the side of the n×n grid, and the number and location of removers are determined by the system requirements, as well. However, there is no RL agent for a remover. This means that a remover’s behavior is determined passively by the adjacent sorter’s behavior.

Parcels are randomly fed into the *n*-grid sortation system through emitters, and routing agents on each sorter decide the routing direction for each parcel on itself at every time step. In a distributed manner, the routing agents try to find the shortest path for a type of parcel from its entry emitter to its destination of a remover. In this paper, a total of six emitters are placed on the two sides of the three-grid system, a group of three emitters facing the other one, and six removers are placed in the same way on the other two sides. Figure 1 depicts a three-grid sortation system, where there are 6 emitters (and 6 emission agents), 9 sorters (and 9 routing agents), and 6 removers.

It is assumed that 1∼6 parcels can be put into the system at the same time. Emission agents control the number of parcels put into the system every time step, in order to suppress congestion that may occur on the nine sorters. While parcels are being classified over the system, a situation may arise where two or more adjacent (emission or routing) agents’ action cannot be performed simultaneously at any time step. For example, there may be a situation where two parcels are simultaneously moved to one sorter by two adjacent routing agents or by adjacent routing and emission agents. This situation is called a collision. Later, we will explain in detail how to resolve such collisions and generate penalties for the agents that caused the collisions. The configured system has just nine sorters, and they act as buffers for parcel flow optimization. Therefore, the small number of sorters increases the risk of congestion or collision during the parcel sorting process.

**Figure 2:**
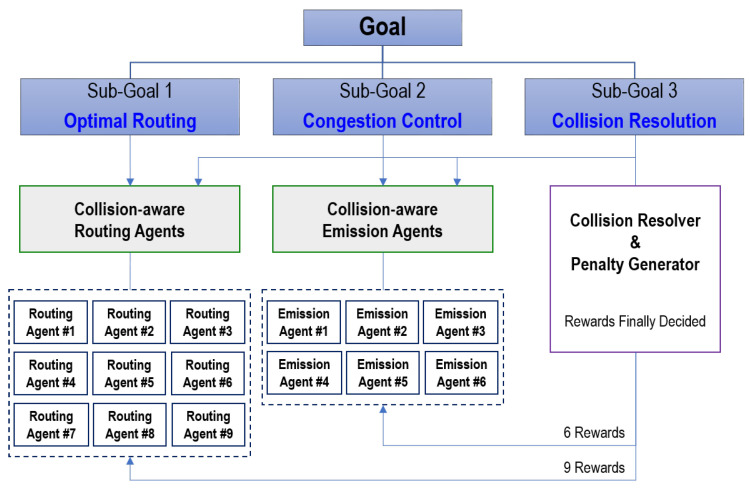
The goal-oriented strategy for cooperative reinforcement learning (RL) multiple agents.

In summary, as depicted in Figure 2, the system consists of three separate sub-goals to achieve the two ultimate objectives: (1) high accuracy and (2) high throughput.

- Optimal routing: sorters should deliver parcels to their specific destination as quickly as possible.
- Congestion control: emitters should control the number of incoming parcels to allow the parcels to be processed and transferred without congestion by the sorters in the system.
- Collision resolution: a system-wide agent should resolve a collision caused by the actions of several routing or emission agents.

## 4. Design of Cooperative the Multi-Agent RL Algorithm

The proposed n×n sortation system should solve the optimization problem for accuracy and throughput. In the system, the routing agents and the emission agents observe the current system state fully at each time step *t* and choose the actions based on their own policy.

**Figure 3:**
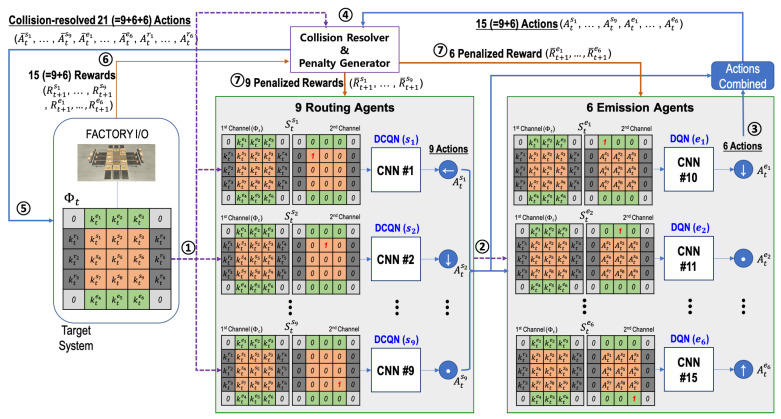
Behavior inference procedure by the cooperative RL multi-agent in the three-grid sortation system.

As shown in Figure 3, for n=3 and each time step *t*, the current system’s state is represented by an 5×5 array, called parcel information $\Phi_{t}$. For a sorter $s_{i}$ (i=1,2,⋯,9) and an emitter $e_{j}$ (j=1,2,⋯,6), the corresponding elements $k_{t}^{s_{i}}$ and $k_{t}^{e_{j}}$ of the array indicate the parcel type (1, 2, or 3 in our case) if a parcel is on the sorter $s_{i}$ or the emitter $e_{j}$, respectively. Otherwise, the corresponding element is set to zero. For a remover $r_{k}$ (k=1,2,⋯,6), the corresponding element $k_{t}^{r_{k}}$ of the array indicates the parcel type (1, 2, or 3) that the remover $r_{k}$ should receive. In a parcel information array $\Phi_{t}$, the pixel values in the four corners are fixed at zero.

### 4.1. Routing Agents

There are nine sorters ($s_{1},s_{2},\cdots ,s_{9}$) in the 3×3 grid system, and the routing agent connected to a sorter controls the sorter’s behavior. In this paper, sometimes, a routing agent will be also symbolized by $s_{i}$, since any routing agent has a one-to-one correspondence with a sorter. For a time step *t*, the state $S_{t}^{s_{i}}$, the action $A_{t}^{s_{i}}$, and the reward $R_{t}^{s_{i}}$ for a routing agent $s_{i}$ (1≤i≤9) are defined as follows.

The state $S_{t}^{s_{i}}$ is an 5×5 two-channel image, where the first channel has the parcel information $\Phi_{t}$. In the second channel, all pixel values are set to zero except for the last pixel where the corresponding sorter $s_{i}$ is located. The value of the last pixel is one. The second channel serves to inform each routing agent of the agent’s location.

The action $A_{t}^{s_{i}}$ represents the selection one of five action types as the sorter $s_{i}$’s action. The five types are “Stop (0)”, “North (1)”, “South (2)”, “West (3)”, and “East (4)”. When a sorter $s_{i}$ performs its action, it will route the parcel to the indicated direction. If the action is zero, the corresponding sorter will do nothing. Therefore, a routing agent will select a non-stop action only when there is a parcel on the corresponding sorter. Before the selected action $A_{t}^{s_{i}}$ is performed in the target system, however, it will be altered to ${\bar{A}}_{t}^{s_{i}}$ by the collision resolver. It should be noted that a sorter can perform the altered non-stop action ${\bar{A}}_{t}^{s_{i}}$ even though there is no parcel on the sorter, because a sorter should perform an action to receive a parcel that an adjacent sorter sends. We will explain this in detail later.

After performing the selected action $A_{t}^{s_{i}}$ at time step *t*, the sorter $s_{i}$ receives a reward $R_{t+1}^{s_{i}}$ at the next time step t+1. It consists of three types of components: the “routing” reward $u_{t+1}^{s_{i}}$, the “correct classification” reward $c_{t+1}$, and the “wrong classification” reward $w_{t+1}$. Each of them has its coefficient and the range of its possible values, which are described in Table 1. The “routing” reward $u_{t+1}^{s_{i}}$ will be one when the sorter $s_{i}$ forwards (i.e., routes) a parcel to an adjacent sorter or remover. A negative “routing” reward coefficient can lead the routing agents to minimize the number of routing behaviors, and thus, the routing path can be shortened. Therefore, this reward design helps increase the throughput measure. The “correct classification” reward $c_{t+1}$ and the “wrong classification” reward $w_{t+1}$ will be set to the number of correctly and wrongly classified parcels (i.e., the number of parcels correctly and wrongly arriving at six removers) at time step t+1, respectively. The two reward values are commonly added into $R_{t+1}^{s_{i}}$ for i=1,⋯,9. It should be noted that the coefficient of the “correct classification” reward is the only positive value. Therefore, this reward design helps increase the accuracy measure. As a result, the reward $R_{t+1}^{s_{i}}$ for the routing agent $s_{i}$ is:

(2) $$\begin{array}{c} R_{t+1}^{s_{i}}=\mu_{u}\cdot u_{t+1}^{s_{i}}+\mu_{c}\cdot c_{t+1}+\mu_{w}\cdot w_{t+1}. \end{array}$$

Before a routing agent $s_{i}$ receives the calculated reward $R_{t+1}^{s_{i}}$, however, the reward will be altered to ${\bar{R}}_{t+1}^{s_{i}}$ by the penalty generator. We will explain it in detail later.

### 4.2. Emission Agents

There are six emitters ($e_{1},e_{2},\cdots ,e_{6}$) in the system, and an emission agent connected to an emitter controls the emitter’s behavior. In this paper, sometimes, an emission agent will be also symbolized by $e_{j}$, since any emission agent has a one-to-one correspondence with an emitter. It should be noted that the action inference of the six emission agents is proceeded after the one of the nine routing agents. For a time step *t*, the state $S_{t}^{e_{j}}$, the action $A_{t}^{e_{j}}$, and the reward $R_{t}^{e_{j}}$ for the emission agent an emission agent $e_{j}$ (1≤j≤6) are defined as follows.

Like the state for the routing agents, the state $S_{t}^{e_{j}}$ is also a 5×5 two-channel image, where the first channel has parcel information $\Phi_{t}$. On the other hand, the second channel consists of the action information inferred by the nine routing agents in the prior phase of the same time step *t*. That is, the nine pixels on the center of the second channel contain $A_{t}^{s_{i}}$ (i=1,2,⋯,9). The 15 pixels on the edge of the second channel are set to zero, and the last pixel corresponding to the emitter’s location has one. The last pixel informs each emission agent of the agent’s location.

The action $A_{t}^{e_{j}}$ corresponds to the selection of one of two action types, “Stop (0)” and “Emission (1)”, as the emitter $e_{j}$’s action. At every time step, six parcels are placed on all six emitters. The type of parcel is randomly determined when placed on an emitter. When an emitter $e_{j}$ performs the action “Emission (1)”, the parcel on the emitter will be moved to the emitter’s adjacent sorter, and a new parcel is immediately placed on the emitter. If the action is “Stop (0)”, the corresponding emitter will do nothing, and the parcel on the emitter will be in place. Before the selected action $A_{t}^{e_{j}}$ (j=1,2,⋯6) is performed in the target system, however, it will be altered to ${\bar{A}}_{t}^{e_{j}}$ by the collision resolver. We will explain this in detail later.

After performing the selected action $A_{t}^{e_{j}}$ at time step *t*, the emitter agent $e_{j}$ receives a reward $R_{t+1}^{e_{j}}$ at the next time step t+1. It consists of two types of components: the “emission” reward $in_{t+1}^{e_{j}}$ and the “in and out balance” reward $balance_{t+1}$. Each of them has its coefficient and the range of its possible values, which are described in Table 1. The “emission” reward $in_{t+1}^{e_{j}}$ indicates whether or not the emitter $e_{j}$ places its parcel on sorters at time step t+1. The corresponding coefficient is positive, which leads the emitters to put as many parcels as possible into the sorters. On the other hand, the “in and out balance” reward $balance_{t+1}$ is determined by the following equation:

(3) $$\begin{array}{cc} balance_{t+1} & =|in_{t+1}-out_{t+1}|\\ \mathrm{where}\;in_{t+1} & =\sum_{j=1,\cdots ,6}in_{t+1}^{e_{j}}\\ out_{t+1} & =\sum_{k=1,\cdots ,6}out_{t+1}^{r_{k}}. \end{array}$$

In Equation (3), $in_{t+1}$ and $out_{t+1}$ indicate the number of parcels fed to sorters by six emitters and the number of parcels arriving at the six removers at time step t+1, respectively. For all j=1,⋯,6, the same value of this reward is commonly added to $R_{t+1}^{e_{j}}$. The corresponding coefficient is negative, which leads the emitters to control the parcel congestion on the sorters. That is, if $balance_{t+1}$ becomes high (e.g., there is no balance between inflow and outflow of parcels), the emitters will reduce or increase the number of parcels newly placed on sorters. Therefore, this reward design helps increase the throughput measure, as well. As a result, the reward $R_{t+1}^{e_{j}}$ for the emission agent $e_{j}$ is:

(4) $$\begin{array}{c} R_{t+1}^{e_{j}}=\mu_{in}\cdot in_{t+1}^{e_{j}}+\mu_{balance}\cdot balance_{t+1}. \end{array}$$

Before the emission agent $e_{j}$ receives the calculated reward $R_{t+1}^{e_{j}}$, however, the reward will be altered to ${\bar{R}}_{t+1}^{e_{j}}$ by the penalty generator. We will explain this in detail later.

### 4.3. Collision Resolver and Penalty Generator

**Figure 4:**
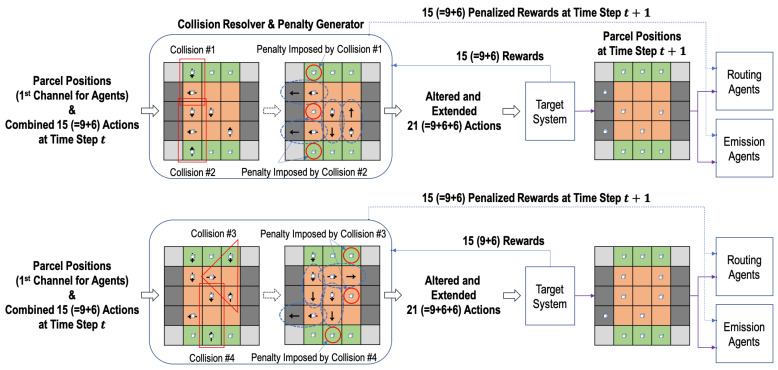
Diverse scenarios of collisions and their resolving in the three-grid sortation system.

In the design of RL agents in the *n*-grid sortation system, a collision occurs when the actions selected by two or more adjacent emission or routing agents cannot be performed at the same time step. Figure 4 describes diverse scenarios of collisions. In the figure, the locations of parcels and the actions of agents in each of the red rectangles or triangle represents a collision. Collisions #1 and #2 in the figure occur because the action directions of two or more routing and emission agents are inconsistent and cannot be performed simultaneously. Collisions #3 and #4 in the figure occur because two or more parcels are supposed to be simultaneously moved to one sorter by two or more routing and emission agents.

With the information of parcel positions and the combined actions at time step *t*, the collision resolver prevents such collisions. Among the actions involved in a collision, it selects the one action that will be performed at time step *t* and alters the rest of actions as “Stop (0)”. The selection rules are simply as follows:

- The action of moving the parcel closer to the remover is selected first,
- The sorter’s action takes precedence over the emitter’s action, and
- If two or more actions have the same priority, one action is randomly selected.

In the first rule, the Manhattan distance is used to measure the distance between a parcel and a remover.

On the other hand, let us assume that an emission or a routing agent α is supposed to perform a non-stop action and move a parcel at time step *t*. Then, the adjacent routing agent receiving the parcel that the agent α moves should perform the action of the same direction as the action’s one of the agent α. Therefore, the routing agent’s action can be altered to a non-stop action by the collision resolver, even though no parcel is located on the agent. Similarly, a remover’s action can be altered to a non-stop action for the remover to receive a parcel from a routing agent (i.e., a sorter). Actions altered in this way are denoted by the dotted ovals in Figure 4. Finally, the collision resolver alters the original actions $A_{t}^{s_{i}}$ and $A_{t}^{e_{i}}$ and produces a total of 21 (=9+6+6) actions, i.e., ${\bar{A}}_{t}^{s_{1}},\cdots ,{\bar{A}}_{t}^{s_{9}}$ for the nine sorters, ${\bar{A}}_{t}^{e_{1}},\cdots ,{\bar{A}}_{t}^{e_{6}}$ for the six emitters, and $A_{t}^{r_{1}},\cdots ,A_{t}^{r_{6}}$ for the six removers.

It should be noted that the routing or emission agents whose actions are altered by the collision resolver to “Stop (0)” will be penalized when they receive the reward from the target system at the next time step. In Figure 4, the agents where the penalty is imposed are denoted by the solid red circles. The original rewards at time step t+1 are first delivered to the penalty generator (co-located with the collision resolver), and the pre-defined penalty values are imposed in the rewards per agent depending on the selection decision of the collision resolver at time step *t*. There are two kinds of collision penalty values, which are presented in Table 1: (1) $p_{t+1}^{s_{i}}$ for a routing agent $s_{i}$ and (2) $p_{t+1}^{e_{j}}$ for an emission agent $e_{j}$. For the two penalty values, the same negative coefficient $\mu_{p}$ is used. Therefore, the original rewards $R_{t+1}^{s_{i}}$ and $R_{t+1}^{e_{j}}$ are changed into ${\bar{R}}_{t+1}^{s_{i}}$ and ${\bar{R}}_{t+1}^{e_{j}}$ as follows:

(5) $$\begin{array}{cc} {\bar{R}}_{t+1}^{s_{i}} & =R_{t+1}^{s_{i}}+\mu_{p}\cdot p_{t+1}^{s_{i}}. \end{array}$$

(6) $$\begin{array}{cc} {\bar{R}}_{t+1}^{e_{j}} & =R_{t+1}^{e_{j}}+\mu_{p}\cdot p_{t+1}^{e_{j}}. \end{array}$$

and the changed ones are delivered to the routing and emission agents at time step t+1.

### 4.4. Cooperative Multi-Agent RL Algorithm

In this section, we describe the proposed multi-agent RL algorithm to optimize the behavior of sorters and emitters in the *n*-grid sortation system. The proposed algorithm is based on DQN, so that the agents have off-policy based learning methods. The algorithm are performed as follows:

(1) The routing agents and the emission agent initialize their experience replay memories, action-value functions ($Q^{s_{i}}$ and $Q^{e_{j}}$), and target action-value functions (${\hat{Q}}^{s_{i}}$ and ${\hat{Q}}^{e_{j}}$) (see Lines 1∼8 of Algorithm 1).
(2) An episode begins with the reset of the target system. At the reset of the target system (that is, time step t=1), each emitter has one parcel of a random type, and no parcel is on a sorter or a remover. The parcel information on the grid system is set to $\Phi_{t}$ (see Lines 10∼11 of Algorithm ① and 1 in Figure 3).
(3) Based on the states configured with $\Phi_{t}$ and the routing agent’s location, the routing agents select their actions according to the ϵ-greedy policy. If there is no parcel on a routing agent, its action is zero indicating “Stop” (see Lines 13∼22 of Algorithm 1 and ② in Figure 3).
(4) Based on the states configured with $\Phi_{t}$ and the actions selected by nine routing agents, the emission agents select their actions according to the ϵ-greedy policy (see Lines 23∼29 of Algorithm 1 and ③ in Figure 3).
(5) The selected actions are delivered to the collision resolver, and it alters the actions according to the rules presented by Section 4.3 (see Line 30∼31 of Algorithm 1 and ④ in Figure 3).
(6) The altered actions at time step *t* are finally performed on the target system, and the rewards at time step t+1 are generated from it (see Lines 32∼33 of Algorithm 1 and ⑤ in Figure 3).
(7) The rewards are also delivered to the penalty generator (co-located with collision resolver), and the penalized rewards are generated by it (see Lines 34∼35 of Algorithm 1 and ⑦ in Figure 3). The penalized rewards will be delivered to the routing and emission agents. However, such delivery is not performed directly at the current time step *t*, but will be performed at the optimization phase of the state-action value functions.
(8) The states, actions, altered actions, penalized rewards, parcel information, and episode ending information are stored in the corresponding replay memory as transition information (see Lines 36∼41 of Algorithm 1). The transition information including penalized rewards will be used when the state-action value functions are optimized. Since the actions selected by routing agents are required for the optimization task of emission agents, $A_{t}^{S}$ is configured and put into the transitions for emission agents.
(9) Model weight copying to the target models is performed every τ episodes (see Line 43 of Algorithm 1).

The optimization is performed on an episode basis, since there is not enough time to optimize their models between time steps in an episode. (Since our target system is configured on Factory I/O (i.e., a virtual simulator), the optimization can be performed even on a step basis. However, the proposed algorithm will be also executed with a real system deployed in a factory. In a real system, there will not be enough time to perform the optimization on a step basis.)

To determine whether an episode ends, two numbers, *L* and *M*, are tracked as follows:

- *L*: the total number of parcels arriving at a remover (i.e., the number of parcels classified) and
- *M*: the number of parcel moves by any one sorter.

In addition, there are two different thresholds $\theta_{L}$ and $\theta_{M}$, and if $L>=\theta_{L}$ or $M>=\theta_{M}$ in the course of an episode, the episode comes to end. When the selected actions are performed at time step *t*, the target system notifies the agents of the end of the episode through the Boolean variable $done_{t+1}$ (see Line 33 of Algorithm 1).
**Algorithm 1:** The proposed multi-agent RL algorithm (Γ: target system, Ω: collision resolver and penalty generator, *ϵ* ∈ [0, 1]: exploration threshold).
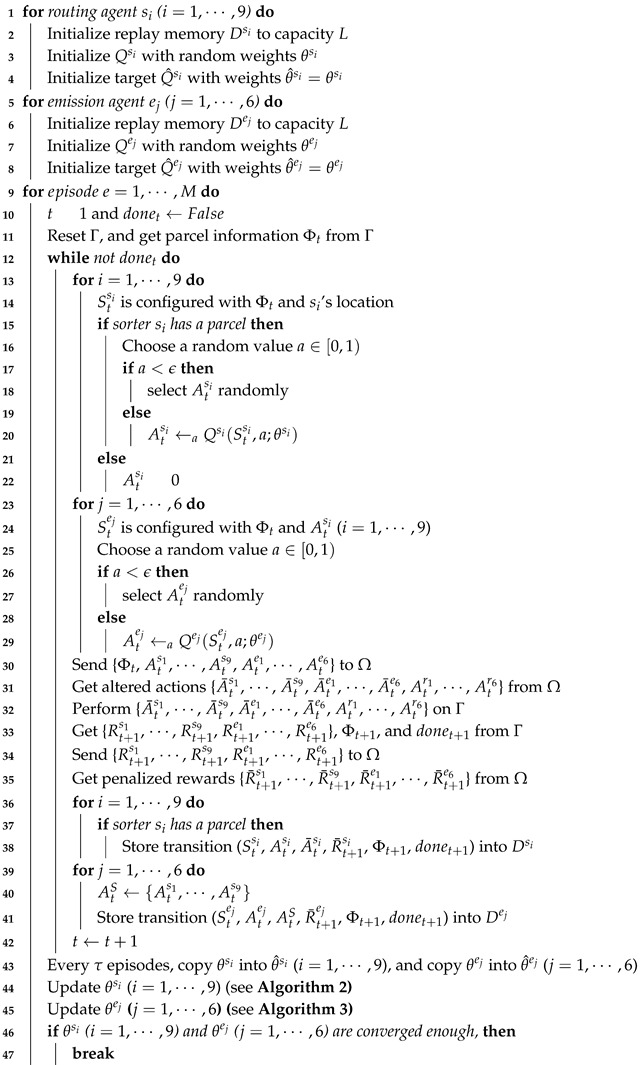


On the other hand, the state-action value function $Q^{s_{i}}$ optimization is performed at each routing agent $s_{i}$ (i=1,⋯,9) as follows:

(1) A routing agent $s_{i}$ samples a random mini-batch of *K* transitions from its replay memory $D^{s_{i}}$ (see Line 2 of Algorithm 2).
(2) For each transition, the routing agent should get the next routing agent or remover η, which its action is bound to, because the target value for the loss equation at time step *t* comes from η (see Line 4 of Algorithm 2).
(3) The action moving a parcel to η was selected by $s_{i}$ according to its policy (i.e., ϵ-greedy at $Q^{s_{i}}$) at the current time step *t*. At time step t+1, after one step progresses, the moving parcel passes to η. Therefore, the target value for the loss equation should be provided by η (see Line 9 of Algorithm 2). We call this strategy the deep chain Q network (DCQN). The strategy is similar to the one of Q-routing [32], which is a well-known RL algorithm in the field of computer network where a node needs to select its adjacent node where the node sends a network packet so that the node delivers the packet to its final destination as soon as possible.

By Algorithm 2, the routing agents learn the optimal routing paths for parcels. However, when routing agents find the optimal routing paths, those may change each time according to the type of parcels and their entry order. Therefore, the routing agents need to collaborate with other agents while predicting future invisible situations. This collaboration is achieved to some extent by the rewards $c_{t+1}$ and $w_{t+1}$ common to all routing agents.
**Algorithm 2:**$Q^{s_{i}}$ optimization for routing agent $s_{i}$ (i=1,⋯,9).
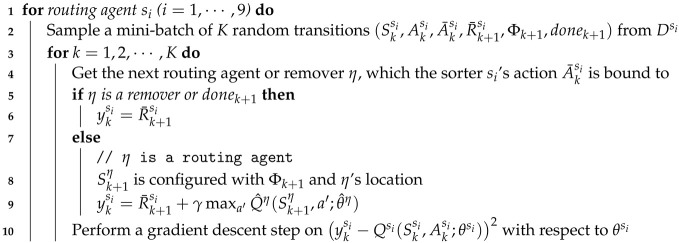


Parcel loads imposed on routing agents are different each time, and congestion can occur on the grid system. Such congestion can be prevented by emission agents. The emission agents adjust the number of the emitted parcels by predicting the overall flow of parcels through the transition information of the mini-batch and the included reward value. The state-action value function $Q^{e_{j}}$ optimization is performed at each emission agent $e_{j}$ (j=1,⋯,6) as follows:

(1) The transitions in a mini-batch are sampled sequentially (see Line 2 of Algorithm 3).
(2) The target value for the loss equation at time step *t* is calculated just from (1) the reward of the next time step t+1 and (2) the maximum state-action value for the emission agent at the next time step t+1 (see Lines 7∼8 of Algorithm 3). For the next time step t+1, $S_{k+1}^{e_{j}}$ should be configured with the actions of the routing actions at time step t+1. Therefore, The transitions in a mini-batch should be sampled sequentially.

In the reward ${\bar{R}}_{k+1}^{e_{j}}$ of each transition, $in_{t+1}^{e_{j}}$ induces an emitter $e_{j}$ to release its parcel into the adjacent sorter. On the other hand, $balance_{t+1}$ is common to all emission agents, and the collaboration between emission agents is achieved to some extent by this reward.
**Algorithm 3:**$Q^{e_{j}}$ optimization for routing agent $e_{j}$ (j=1,⋯,6).
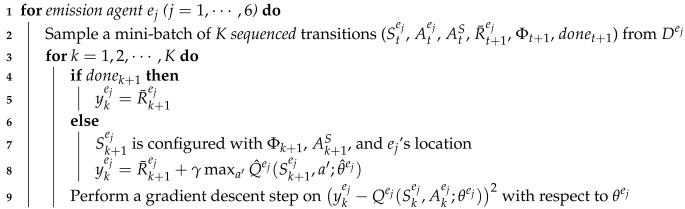


## 5. Performance Evaluation

In this section, we evaluate the performance of the proposed multi-agent RL algorithm on the three-grid sortation system implemented on Factory I/O. We also present that the well-trained collaborative RL agents could optimize their performance effectively.

### 5.1. Training Details and Performance Measure

We performed our experiments on the three-grid sortation system, the configuration of which is explained in Figure 1 in Section 3. In the target system, with the collision resolver, the proposed two types of agents worked together to sort and move the parcels as fast as possible and prevent parcel congestion through continuous learning. Training was performed on an episode basis, and during an episode, an unlimited number of parcels of three types were fed by the emitters at random. However, we set $\theta_{L}$ to 512, so that an episode came to end when 512 parcels were classified on removers. We also set $\theta_{M}$ to 1024, so that an episode also came to end when any one sorter moved parcels more than 1024 times. At this time, all the sorters obtained much negative routing reward $u_{t}^{s_{i}}$, so that it could prevent two or more sorters from sending and receiving the same parcels many times.

In this study, a new performance indicator, called the sorting performance index (SPI), is presented to measure how well the routing and emission agents achieved the two given goals, high accuracy and high throughput, together. For an episode *e*, $SPI_{e}$ is given by the following equation:

(7) $$\begin{array}{cc} SPI_{e} & =\frac{C_{e}-W_{e}}{\mathrm{the}\;\mathrm{number}\;\mathrm{of}\;\mathrm{removers}\;\left(=6\right)}\\ \mathrm{where}\;C_{e} & =\frac{\sum_{t=1}^{T_{e}}c_{t}}{T_{e}}\;\mathrm{and}\;W_{e}=\frac{\sum_{t=1}^{T_{e}}w_{t}}{T_{e}} \end{array}$$

where $T_{e}$ indicates the total time steps elapsed during episode *e*. The two values $c_{t}$ and $w_{t}$ represent the numbers of parcels classified correctly and wrongly at time step *t*, respectively ($0\leq c_{t}+w_{t}\leq \mathrm{the}\;\mathrm{number}\;\mathrm{of}\;\mathrm{removers}\;\left(=6\right))$. As shown in Table 1, the two values are components of the reward delivered to a routing agent, and by using Equation (3), $out_{t}=c_{t}+w_{t}$. Therefore, $C_{e}$ indicates the average correct classification rate per time step, while $W_{e}$ indicates the average wrong classification rate per time step. The SPI value was obtained by the difference between the two rates for an episode. It was also divided by the number of removers (=6) for normalization, so that it was between −1.0 and 1.0.

In the learning experiment, a total of 2700 episodes were performed repeatedly. We used ϵ-greedy as the behavior sampling policy for routing and emission agents, and the ϵ value was gradually decreased from 0.5 to 0.0 across all the episodes. Finally, the step size (learning rate) α was set to 0.0001, the discount factor γ to 0.99, and the episode interval for weight copying τ to five. It took five hours to finish the learning experiment on the GEFORCE RTX 2080 Ti GPU. We conducted seven experiments and collected the average, the maximum, and the minimum values of the performance measures for every episode.

### 5.2. Results

**Figure 5:**
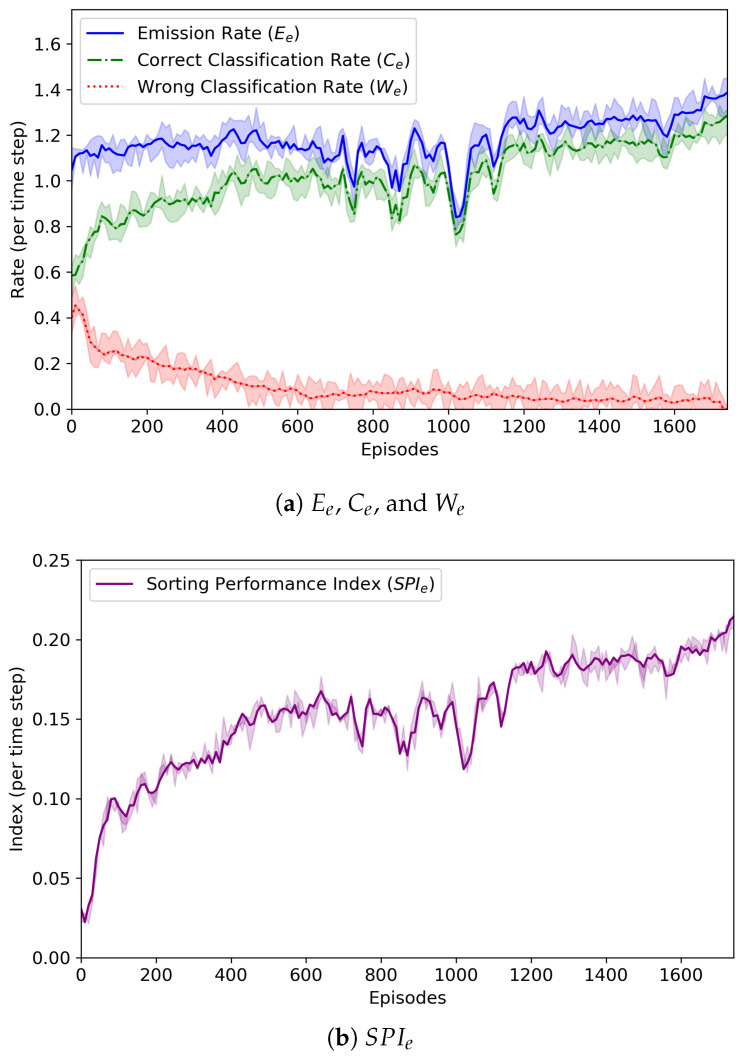
The change of average emission rate ($E_{e}$), average correct classification rate ($C_{e}$), average wrong classification rate ($W_{e}$), and sorting performance index ($SPI_{e}$). We conducted seven experimental runs. The graphs include the lines plotted for the average values of the four measures, while the shaded areas of the upper and lower limits are the maximum and minimum values for the same measures.

Figure 5 reveals the change of the average emission rate $E_{e}$, average correct classification rate $C_{e}$, average wrong classification rate $W_{e}$, and sorting performance index $SPI_{e}$ for each episode *e*. By using Equation (3), the emission rate $E_{e}$ is simply defined as:

(8) $$E_{e}=\frac{\sum_{t=1}^{T_{e}}in_{t}}{T_{e}}.$$

It was found that the emission rate did not become high (it was up to 1.4). The emission agents continuously monitored the behavior of the routing agents, analyzed the given rewards, and controlled the amount of parcels fed to sorters. Since there were just nine sorters in the form of a 3 × 3 grid, a large number of parcels could not be fed into the sorters. From the low emission rate, we could know that the emission agents were trained well and their behavior was appropriate.

The emission agents also tried to balance the number of parcels fed to the sorters with the number of parcels removed by the removers. That is, they tried to make the two values, $E_{e}$ and $C_{e}+W_{e}$, equal. Overall, $C_{e}+W_{e}$ was lower than $E_{e}$. This was because if $\theta_{L}$ (=512) parcels were classified by agents or $\theta_{M}$ (=1024) times of moves were performed by a sorter, an episode came to end even though a number of unclassified parcels still remained on the sorters.

As shown in Figure 5a, at the beginning of the learning experiment, $C_{e}$ and $W_{e}$ were almost similar, but as learning was repeated, $W_{e}$ tended to decrease and $C_{e}$ to increase. From these results, we could know that the routing agents worked together to find the correct routing paths for the parcels and the emission agents had the ability to control the number of parcels fed by themselves well.

In Figure 5b, the change of $SPI_{e}$ is also depicted. At the 1780th episode, $SPI_{e}$ was calculated as the largest value, 0.21. It is noted that $W_{e}$ was almost 0.0 at that episode. Whenever the $SPI_{e}$ value hit the highest point, the RL policy model being trained was saved, and we finally obtained the best models of agents. These results proved that the proposed cooperative multi-agent RL algorithm was effective for the *n*-grid sortation system to sort the parcels as fast as possible.

**Figure 6:**
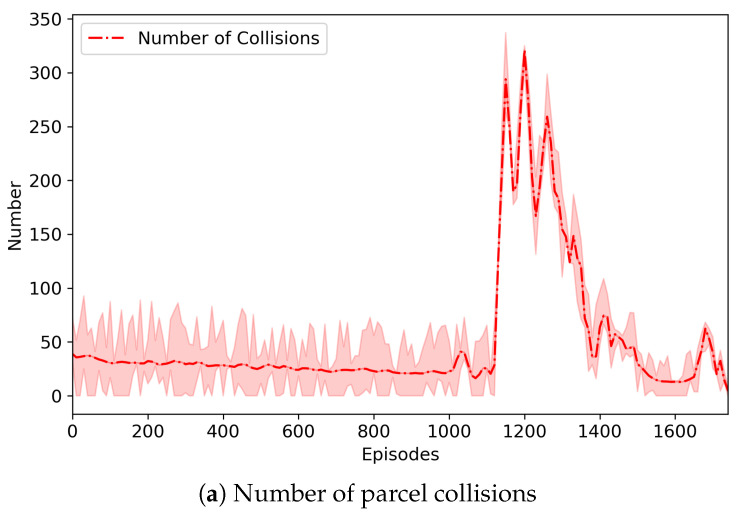

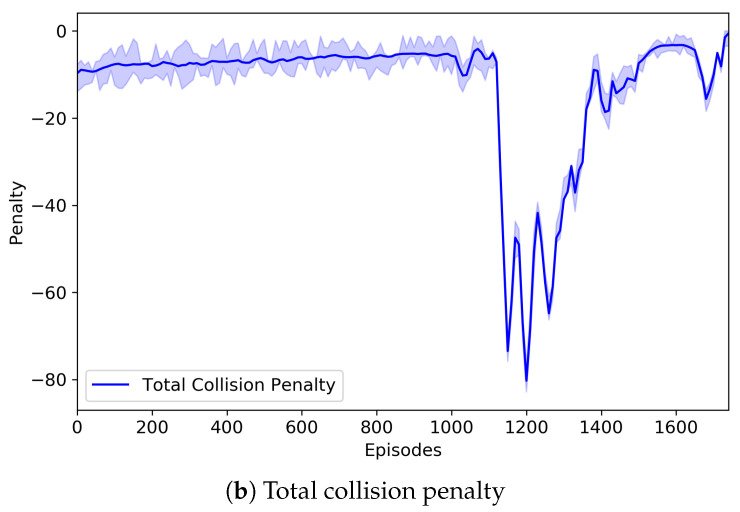
The change of the number of parcel collisions and the change of the total collision penalty imposed on the emission agents. We conducted seven experimental runs. The graphs show the average, the maximum, and the minimum of the two measures like Figure 5.

Figure 6 depicts the change of the number of parcel collisions that may occur in our three-grid sortation system and also represents the change of the total collision penalty imposed on routing and emission agents. In the system, parcels were generated randomly regardless of their type and order, and they should be classified to their designated removers. However, the number of sorters was relatively small, so that there were not enough buffers where parcels moved and were located. This directly led to numerous collisions (or congestion) of parcels. Through repeated learning, however, routing agents were increasingly good at finding the optimal route for parcels, and the emission agents were also increasingly good at controlling the congestion. Nevertheless, routing and emission agents may select their actions that can cause collisions of parcels. In the proposed system, the collision resolver changed them, so that the collision-resolved actions were finally performed on the system. Instead, the collision penalties were imposed as penalized rewards on the routing and emission agents causing the collisions. According to our experiments, the number of collisions varied largely during some episodes, but after those, the number of collisions became very low. It can be seen from Figure 6 that a similar situation occurred. The number of collisions began to vary from about 1000 episodes, and thus, the total collision penalty was changed accordingly. It is noted that the episode (i.e., the 1780th episode) where the best model was saved was the one where the number of collisions almost became zero after the number of collisions was varied.

**Figure 7:**
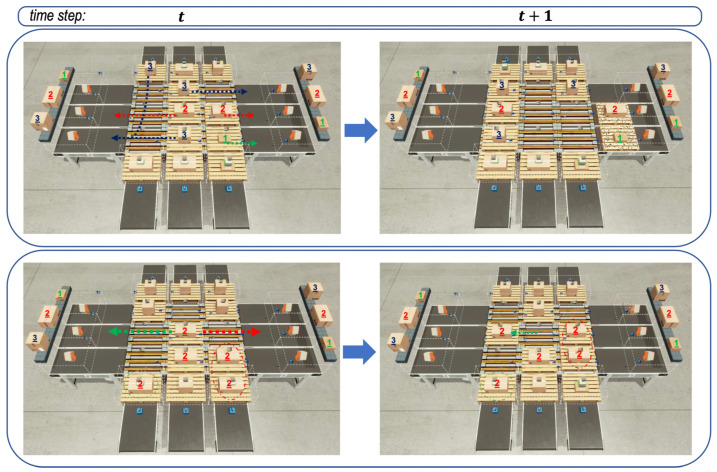
The graph above shows simulated screen captures of the three-grid sortation system controlled by the saved models leading to the emergent behaviors of the agents. In the first sequence of two images at the top, routing and emission agents seemed to consider the location of the parcels placed on different agents and increased throughput with unobtrusive behavior. In the second sequence of two images at the bottom, there were many parcels of the same type on the routing and emission agents, and there were just two removers for the type of parcels. In such a situation, agents seemed to perform routing so that there was less congestion.

Finally, Figure 7 depicts the behavior of emitters and sorters that were controlled by the well-trained RL agents. In fact, the well-trained RL agents had the models saved at the 1780th episode. The figure shows the behaviors between two successive time steps *t* and t+1 on the left and right sub-figures, so that we can try to expect the next behaviors of emitters and sorters from the left sub-figure and check the behaviors selected by emitters and sorters from the right sub-figure. By expecting and checking from two sub-figures, we could know that the agents performed their actions well, as expected.

## 6. Conclusions

In this paper, we designed an *n*-grid sortation system that could be used in the intralogistics industry and developed a collaborative multi-agent RL algorithm to control the behavior of emitters or sorters in the system. The system’s goal had two sub-goals: (1) high accuracy and (2) high throughput; that is, the parcels fed into sorters should be routed to the removers correctly and quickly. There were two types of RL agents, emission agents and routing agents, and they were trained to solve the given multi-objective optimization problem together. We described the agents’ learning performance by depicting the changes of the SPI value, as well as the emission rate, the correct classification rate, and the wrong classification rate. We also represented the changes of the number of parcel collisions and of the total collision penalty imposed on routing and emission agents. The RL models of agents were saved whenever the SPI value hit the highest point, so that we could get the best models after the RL learning was over. From the learning results, we could know that the well-trained routing and emission agents could achieve the given multiple objectives effectively. In particular, the routing agents finally learned to route the parcels through their optimal path, and the emission agents finally learned to control the parcel congestion on the sorters.

In future work, we will design a more flexible sortation system so that the formation of the system is not limited to a grid shape. The routing and emission agents should be designed again to support such a flexible formation. In addition to that, we will provide a new design of the RL agents to allow them to resolve the parcel collisions by themselves without the aid of the independent collision resolver.

## Figures and Tables

**Table 1:** The components of rewards at time step t+1 caused by the actions performed by the routing agents and the emission agents at time step *t*.

Symbol	Type	Coefficient	Possible Values
$u_{t+1}^{s_{i}}$	routing	$\mu_{u}=-0.1$	0 or 1
$c_{t+1}$	correct classification	$\mu_{c}=1$	0,1,⋯,6
$w_{t+1}$	wrong classification	$\mu_{w}=-1$	0,1,⋯,6
$p_{t+1}^{s_{i}}$	collision penalty	$\mu_{p}=-0.1$	0 or 1
$in_{t+1}^{e_{j}}$	emission	$\mu_{in}=0.1$	0 or 1
$balance_{t+1}$	in and out balance	$\mu_{balance}=-1$	0,1,⋯,6
$p_{t+1}^{e_{j}}$	collision penalty	$\mu_{p}=-0.1$	0 or 1
